# Isolation and Molecular Characterization of a Novel Picornavirus from Baitfish in the USA

**DOI:** 10.1371/journal.pone.0087593

**Published:** 2014-02-21

**Authors:** Nicholas B. D. Phelps, Sunil K. Mor, Anibal G. Armien, William Batts, Andrew E. Goodwin, Lacey Hopper, Rebekah McCann, Terry Fei Fan Ng, Corey Puzach, Thomas B. Waltzek, Eric Delwart, James Winton, Sagar M. Goyal

**Affiliations:** 1 Minnesota Veterinary Diagnostic Laboratory, St. Paul, Minnesota, United States of America; 2 University of Minnesota, Department of Veterinary Population Medicine, St. Paul, Minnesota, United States of America; 3 U.S. Geological Survey, Western Fisheries Research Center, Seattle, Washington, United States of America; 4 U.S. Fish and Wildlife Service, Portland, Oregon, United States of America; 5 U.S. Fish and Wildlife Service, Bozeman Fish Health Center, Bozeman, Montana, United States of America; 6 U.S. Fish and Wildlife Service, La Crosse Fish Health Center, Onalaska, Wisconsin, United States of America; 7 Blood Systems Research Institute, San Francisco, California, United States of America; 8 University of California, Department of Laboratory Medicine, San Francisco, California, United States of America; 9 University of Florida, College of Veterinary Medicine, Department of Infectious Diseases and Pathology, University of Florida, Gainesville, Florida, United States of America; Columbia University, United States of America

## Abstract

During both regulatory and routine surveillance sampling of baitfish from the states of Illinois, Minnesota, Montana, and Wisconsin, USA, isolates (n = 20) of a previously unknown picornavirus were obtained from kidney/spleen or entire viscera of fathead minnows (*Pimephales promelas*) and brassy minnows (*Hybognathus hankinsoni*). Following the appearance of a diffuse cytopathic effect, examination of cell culture supernatant by negative contrast electron microscopy revealed the presence of small, round virus particles (∼30–32 nm), with picornavirus-like morphology. Amplification and sequence analysis of viral RNA identified the agent as a novel member of the *Picornaviridae* family, tentatively named fathead minnow picornavirus (FHMPV). The full FHMPV genome consisted of 7834 nucleotides. Phylogenetic analysis based on 491 amino acid residues of the 3D gene showed 98.6% to 100% identity among the 20 isolates of FHMPV compared in this study while only 49.5% identity with its nearest neighbor, the bluegill picornavirus (BGPV) isolated from bluegill (*Lepomis macrochirus*). Based on complete polyprotein analysis, the FHMPV shared 58% (P1), 33% (P2) and 43% (P3) amino acid identities with BGPV and shared less than 40% amino acid identity with all other picornaviruses. Hence, we propose the creation of a new genus (*Piscevirus*) within the *Picornaviridae* family. The impact of FHMPV on the health of fish populations is unknown at present.

## Introduction

Baitfish are economically and ecologically important throughout many regions of the world [1]. For example in the USA, 257 baitfish farms produced $38 million worth of baitfish in 2005, which ranks in the top five for aquaculture production [2]. In addition, a relatively undocumented, but significant sector of the baitfish industry relies upon fish harvested from the wild [3]. The most important baitfish species in the USA are the golden shiner (*Notemigonus crysoleucas*) and the fathead minnow (*Pimephales promelas*), although a variety of other wild and farmed fish species are also produced [4]. The baitfish industry in the USA ships more than 10 billion fish per year across state lines and nationwide. These fish are distributed through wholesale and retail networks to anglers who take the live baitfish to rivers and lakes where they may be consumed by predators or released into the wild [5]. In addition, large quantities of baitfish are sold as forage for the production of predatory species such as walleye (*Sander vitreus*) and muskellunge (*Esox masquinongy*).

The movement of baitfish into new watersheds could potentially introduce viruses from an endemic area, where fish have co-evolved with a particular pathogen, to new bodies of water where fish hosts may have little or no natural resistance [5]–[8]. Regulatory oversight of these movements has become increasingly strict with the emergence of high profile pathogens, such as viral hemorrhagic septicemia virus (VHSV) in the Great Lakes [9], [10]. Currently, many state, national, and international regulations mandate the inspection of certain aquatic animal species prior to movement using standard methods [11], [12]. Such methods often specify virus isolation in cell culture as the gold standard diagnostic test for the detection of fish viruses.

The isolation of a virus in cell cultures is largely non-specific unless followed by virus identification and characterization. It is not surprising then, that due to the increased testing of baitfish and the use of cell culture-based diagnostic assays, reports of novel viral pathogens of fish are becoming more common. This was demonstrated during a 2009–2011 survey of Wisconsin baitfish dealers in which McCann [13] isolated one or more viruses in 36 of 82 lots inspected, resulting in a total of 39 viral isolates. The isolation of previously known aquareoviruses (51%) and nidoviruses (10%) was not surprising; however, 39% of the isolates could not be easily characterized and thus, cause for concern [13]. Simultaneously in other laboratories, previously unknown viruses were isolated from fathead minnows from Illinois, Minnesota, Montana, and Wisconsin, and brassy minnows (*Hybognathus hankinsoni*) from Montana. Over the course of time it became apparent that many of the aforementioned isolates were related to each other and were putative members of the *Picornaviridae* family.

The *Picornaviridae* family is currently divided into 17 genera: Aphthovirus, Aquamavirus, Avihepatovirus, Cardiovirus, Cosovirus, Dicipivirus, Enterovirus, Erbovirus, Hepatovirus, Kobuvirus, Megavirus, Parechovirus, Salivirus, Sapelovirus, Senecavirus, Teschovirus and Tremovirus [14], [15], however the list is rapidly expanding (www.picornaviridae.com). Picornaviruses are small (∼30–32 nm), icosahedral, non-enveloped single stranded positive sense RNA viruses with genome size of approximately 7.2 to 9.0 kb [14]. The genome encodes a single polyprotein flanked by 5′ and 3′ nontranslated regions (NTRs). The viral polyprotein is post-translationally cleaved into three regions P1, P2 and P3. These three regions are further processed into 10–12 small viral proteins, such as viral capsid proteins (VP4, VP3, VP2, VP1), which are encoded by P1 while P2 and P3 encode non-structural proteins that facilitate protein processing (2A^pro^, 3C^pro^ and 3CD^pro^) and genome replication (2B, 2C, 3AB, 3B (VPg), 3CD^pro^, 3D^pol^) [16]. In addition to these proteins, the picornaviruses in some genera also contain a leader protein (L) upstream of the P1.

Picorna-like viruses have been reported sporadically in various fish species [17]–[20], although some of these were later shown to be members of other virus families [21], [22]. From mortality events of bluegill (*Lepomis macrochirus*) in Montana Lake in northeastern Wisconsin, a picornavirus, tentatively known as the bluegill picornavirus (BGPV), was recently isolated and molecularly characterized [23]. Phylogenetic analysis of the BGPV genome (GenBank NC_018506) revealed the virus to be the member of a new genus in the family *Picornaviridae* [23].

Herein, we describe the isolation and characterization of a novel picornavirus from baitfish and compare multiple isolates obtained from various laboratories to determine the relationship of the fathead minnow picornavirus (FHMPV) with previously reported picornaviruses and discuss the implications of these findings.

## Materials and Methods

### Source of Samples

A total of 20, previously uncharacterized, viral isolates were included in this study (Table 1). Eight of these isolates (FHMPV-01 thru FHMPV-08) were cultured at the La Crosse Fish Health Center (U.S. Fish and Wildlife Service, Onalaska, WI) as a result of mandatory inspections of four Wisconsin baitfish dealers [13]. The isolates originated from fathead minnows, collected from licensed aquaculture facilities in Minnesota and Wisconsin. However, these facilities commonly import baitfish from a variety of sources in and out of state, combining them for later resale. Consequently, the precise source populations are unknown.

**Table 1:** Fathead minnow picornavirus isolates included in this study. FHM = fathead minnow, BRM = brassy minnow, R. = reservoir.

Isolate ID	Host	Sample Date	Source Location	State	Cell Line	Incubation Temperature	GenBank Accession Number
FHMPV-1	FHM	May 2010	Baitfish Wholesaler	MN	EPC	20°C	KF183915
FHMPV-2	FHM	April 2010	Baitfish Wholesaler	MN	EPC	20°C	KF183916
FHMPV-3	FHM	June 2010	Baitfish Wholesaler	MN	EPC, CHSE	20, 15°C	KF183909
FHMPV-4	FHM	June 2010	Baitfish Wholesaler	MN	EPC, CHSE	20, 15°C	KF183910
FHMPV-5	FHM	Nov 2010	Baitfish Wholesaler	WI	EPC	20°C	KF183911
FHMPV-6	FHM	Dec 2011	Baitfish Wholesaler	MN	EPC	20°C	KF183912
FHMPV-7	FHM	Dec 2011	Baitfish Wholesaler	MN	EPC	20°C	KF183913
FHMPV-8	FHM	Dec 2011	Baitfish Wholesaler	MN	EPC	20°C	KF183914
FHMPV-9	FHM	Oct 2009	Gullwing R.	MT	EPC, BF-2	22°C	KC465953
FHMPV-10	FHM	June 2006	Gullwing R.	MT	EPC, BF-2	22°C	KF824535
FHMPV-11	FHM	June 2006	Gullwing R.	MT	EPC, BF-2	22°C	KF824536
FHMPV-12	FHM	June 2006	Gullwing R.	MT	EPC, BF-2	22°C	KF824537
FHMPV-13	FHM	Oct 2009	Desert Coulee R.	MT	EPC, BF-2	22°C	KF824538
FHMPV-14	FHM	Oct 2009	Desert Coulee R.	MT	EPC, BF-2	22°C	KF824539
FHMPV-15	FHM	July 2011	Compton R.	MT	EPC, BF-2	22°C	KF824540
FHMPV-16	FHM	July 2011	Bison Bone R.	MT	EPC, BF-2	22°C	KF824541
FHMPV-17	BRM	July 2011	Anderson R.	MT	EPC, BF-2	22°C	KF824542
FHMPV-18	BRM	July 2011	Anderson R.	MT	EPC, BF-2	22°C	KF824543
FHMPV-19	FHM	July 2011	Anderson R.	MT	EPC, BF-2	22°C	KF824544
FHMPV-20	FHM	May 2003	Farm	IL	RTG-2, FHM	15°C	KF874490

Eleven viral isolates (FHMPV-09 thru FHMPV-19) cultured at the Bozeman Fish Health Center (U.S. Fish and Wildlife Service, Bozeman, MT) were also included in this study. The isolates originated from fathead minnows and brassy minnows collected from five different bodies of water in Montana as part of a routine wild fish health survey.

One viral isolate (FHMPV-20) cultured at the University of Arkansas at Pine Bluff, Fish Disease Laboratory (Pine Bluff, AR) was also included in this study. The isolate originated from apparently healthy fathead minnows during a routine farm inspection. The source population was a fathead minnow farm in Illinois.

### Virus Isolation

Homogenates of kidney/spleen or entire viscera were inoculated onto either the *epithelioma papulosum cyprini* (EPC), fathead minnow (FHM), Chinook salmon embryo (CHSE-214), bluegill fry (BF-2), or rainbow trout gonad (RTG-2) cell lines at 15–22°C using standard methods [11]. Infected cell cultures exhibiting CPE were subjected to additional procedures for virus identification and characterization. Briefly, the cell culture suspension was centrifuged at 1,500×g for 15 min and the supernatant collected. The RNA and DNA were extracted using the QIAamp viral RNA mini kit or the DNeasy blood and tissue kit following the manufacturer’s recommendation (Qiagen). Isolates were tested by polymerase chain reaction (PCR) or reverse transcription-polymerase chain reaction (RT-PCR) assays according to [11], unless otherwise noted for common and/or reportable fish pathogens, such as VHSV, spring viremia of carp virus (SVCV), infectious pancreatic necrosis virus (IPNV), largemouth bass virus (LMBV), bluegill picornavirus (BGPV) [23], golden shiner virus (GSV) [24], and fathead minnow nidovirus (FHMNV) [25]. Cultures for which a virus could not be identified were also subjected to negative contrast electron microscopy (EM) for morphologic characterization and to various PCR amplification strategies to obtain authentic sequences for molecular analysis.

### Electron Microscopy

The culture supernatant from infected EPC cells (the FHMPV-01 isolate) was centrifuged at 2,900×g for 10 min followed by centrifugation at 30 PSI using an airfuge (Beckman Coulter) for 10 min. The supernatant from the final spin was discarded and the pellet was re-constituted in 10 µl of double distilled water. The suspension was placed on formvar-coated copper grids (Electron Microscopy Science) and stained with 1% phosphotungstic acid (Electron Microscopy Sciences) for 1 min. The grids were observed under a JEOL 1200 EX II transmission electron microscope (JEOL LTD). The images were obtained using a Veleta 2K × 2K camera with iTEM software (Olympus SIS).

### Partial Genome Sequencing: FHMPV-01 to FHMPV-08

Genome sequences of FHMPV-01 to -08 were analyzed by the Minnesota Veterinary Diagnostic Laboratory (St. Paul, MN). For preliminary identification of the FHMPV-01, RNA was extracted from infected and mock-inoculated cell culture supernatants by using a viral RNA mini kit (Qiagen). cDNA was synthesized using the superscript III RT kit (Invitrogen) and Oligo (dT)_20_ supplied with the kit. The PCR reaction was carried out on amplified cDNA by using universal primer 5-CCGACTCGAG*INNNNNN*TGTGG-3 [26]. The amplified products were run on 1.2% agarose gel, purified using Qiagen PCR purification kit, and then cloned with Zero blunt PCR cloning kit (Invitrogen). Insertion was confirmed by running colony PCR with M13 primers (M13 Forward-5′-GTAAAACGACGGCCAG-3′ and M13 Reverse-5′-CAGGAAACAGCTATGAC-3′) as per the protocol given in the Zero blunt PCR cloning kit (Invitrogen). The plasmid was purified from colonies with the insert using the Qiagen miniprep kit followed by sequencing at the University of Minnesota Genomic Center (UMGC) using both forward and reverse M13 primers.

Following the preliminary classification of the novel virus as a picornavirus, additional molecular analysis was performed. For FHMPV-01 and -02, the nearly complete genome sequence was obtained by primer walking. Sequences obtained from cloning matched with different segments of picornaviruses (P1, P2, P3). Primers were designed to fill the gap between sequences of different regions. For the 5′ NTR region, universal primers targeting the 5′ NTR of picornaviruses were used [27]. For the 3′ NTR, Oligo (dT)_20_ was used as the reverse primer while a forward primer was designed from sequence we obtained for the P3 region. Further confirmation of the consensus sequence was achieved by specific FHMPV primers designed to amplify the nearly complete genome. The resulting sequence contained 500 nt of the 5′ NTR and complete polyprotein ORF and entire 3′ NTR. For isolates FHMPV-03 thru -08, the entire 3D gene was sequenced using specific primers. Amplified PCR products were purified using Qiagen PCR purification kit (Qiagen) and then submitted for sequencing with both forward and reverse primers. Forward and reverse sequences were aligned using Sequencher 5.1 software (www.genecodes.com) followed by BLASTx analysis (www.ncbi.nlm.nih.gov) to generate consensus sequences and to perform phylogenetic analysis.

### Partial Genome Sequencing: FHMPV-09 to FHMPV-19

Viral RNA was extracted from isolates by TriReagent (Sigma) with yeast tRNA carrier added for the RNA precipitation step according to the manufacturer’s protocol. Two large genomic regions of 11 FHMPV isolates (FHMPV-09 thru FHMPV-19) were analyzed by the Western Fisheries Research Center (Seattle, WA). The first region, designed to amplify the 3′ end of the polyprotein ORF including the conserved RNA polymerase region, was amplified using primers 5′-GAAAATCTCACCAAAGGAGATTA-3′ and 5′-TTTGGGAAAACATTACACTAAAC-3′, resulting in 2047 nt of sequence. This portion, called 56–61, aligned with the genome sequence of BGPV beginning at nt 6094 to include the end of the 3C proteinase, the entire 3D gene (RdRp), and continuing to also include about 300 nt of the 3′ NTR. The second region, designed to provide gene sequences for several structural proteins, was amplified using 5′-CGGATCAAGAGACCTGTTTTC-3′ and 5′-CAAACGTTGCACCCACCAA-3′, resulting in 1911 nt sequence. This portion called 73–89 aligns with the BGPV at nt 2266–4284 and includes most of the VP1, 2A1, 2A2, 2B, and some of the 2C regions. To amplify these longer RT-PCR reactions, high fidelity Platinum Taq (Invitrogen) was used. The 56–61 region was submitted to GenBank with accession numbers KF824535– KF824544 and region 73–89 with accession numbers KF824545– KF824555. The 56–61 region was used for sequence comparison and phylogenetic analysis.

### Partial Genome Sequencing: FHMPV-20

FHMPV-20 was sequenced using a deep sequencing approach by the Blood Systems Research Institute (San Francisco, CA) and the University of Florida Wildlife and Aquatic Animal Veterinary Disease Laboratory (Gainesville, Florida). Briefly, virus particles were purified from culture supernatant by filtration and nuclease treatment. Purified viral RNA was extracted and underwent random PCR amplification as previously described [28], [29]. The randomly amplified library was quantified and sequenced using the 454 GS FLX Titanium platform (Roche). The resulting 454 pyrosequences were trimmed for quality and primers, and assembled *de novo* into contigs. Assembled contigs were compared to the GenBank non-redundant protein database using BLASTx with an E-value cutoff of 10^−4^. A near-complete genome of FHMPV-20, including the entire polyprotein coding region, was identified in the assembled data.

### Full Genome Sequencing: FHMPV-09

For full characterization, the entire genome of FHMPV-09 isolated from fathead minnows in Gulling Reservoir, Montana was sequenced by the Western Fisheries Research Center. Because the taxonomic affiliation of FHMPV was initially unknown, a degenerate set of primers was designed based on the nucleotide sequence of segment 10 of a fish aquareovirus. These aquareovirus primers, 5′-ATTCATCCMACTATYGCKACTCA-3′ and 5′-GGCATGGCRTCWGTCTGRACGAT-3′, amplified a 340 bp amplicon from the RNA sample, which was suitable for sequencing using Big Dye chemistry and a 3130 Genetic Analyzer (Applied Biosystems). The sequence showed the highest identity to that of duck hepatitis A virus 1 by BLASTx search (up to 34% amino acid identity). Thus, new sets of PCR primers were made using this authentic sequence and the full genome was obtained by a combination of primer walking and 3′ and 5′ RACE. For the 3′ and 5′ RACE, specific primers were designed to bind genomic RNA sequences of the virus according to the manufacturer′s protocol (Life Technologies). Another primer 5′-TTGAAAGAGAGTCCATACGG-3′ was designed to bind to the 5′ end of the genome allowing confirmation of the 5′ NTR. Additional repeat sequence confirmations were obtained by either amplifying genomic RNA by RT-PCR for small segments of the genome or by using high fidelity Platinum Taq (Invitrogen) to amplify the entire polyprotein open reading frame. Numerous primers were used to obtain adequate sequence coverage of the genome in both directions. Due to a relatively high level of sequence heterogeneity that was characterized by the presence of both a major and a minor peak in both directions of the sequence chromatogram at specific nucleotide positions, additional PCR amplifications were performed with subsequent sequence reactions to confirm the consensus sequence.

Further predictions of structural and non-structural proteins were done based on amino acid alignments with reference picornavirus sequences from Genbank and the presence of cleavage sites identified using the NetPicoRNA program [30]. Cleavage sites at the interdomain junctions were predicted based on the preference of picornaviruses for glutamine (Q) and glutamic acid (E) at the P1 position of the cleavage site (P3-P2-P1*P1’-P2’-P3’, where cleavage is between P1 and P1’) and a small amino acid residue (e.g., glycine (G), serine (S), arginine (R), methionine (M), alanine (A) and asparagine (N)) at the P1’ position [30].

### Sequence Comparisons and Phylogenetic Analysis

The nucleotide sequences obtained were converted into amino acid sequences and aligned with picornavirus sequences available in Genbank (Table 2) by using ClustalW [31] in MEGA 5.2.1 [32]. The evolutionary distances were computed using the Maximum Likelihood Method. The selection of protein evolution model was done by using ProtTest [33] in Phylemon 2.0 [34] and found JTT+G+I (JTT-Jones-Taylor-Thornton, G: Gamma, I: Invariable) as best fit on sorting models based on AIC score. A phylogenetic tree of aligned amino acid sequences was constructed using the best fit model JTT [35]. A discrete Gamma distribution was used to model evolutionary rate differences among sites (5 categories, +G). A rate variation model allowed some sites to be evolutionary invariable (+I). The 1000 bootstrap replicates were used for statistical validation of the phylogenetic tree [36]. Amino acid profile and identity figure of complete ORF comparison was generated by using Geneious Pro [37]. Pairwise comparisons were performed using Species Demarcation Tool [38].

**Table 2:** Related members of the *Picornaviridae* family used for phylogenetic analysis.

Sequence ID	GenBank Accession	Virus name	Genus name
1.	YP_003355055.1	Bovine rhinitis B virus	Apthovirus
2.	NP_937967.1	Foot-and-mouth disease virus - type Asia 1	
3.	NP_740383.1	Equine rhinitis A virus	
4.	NP_740571.1	Equine rhinitis B virus 2	
5.	NP_740368.1	Equine rhinitis B virus 1	
6.	YP_002956087.1	Human cosavirus E	Cosavirus
7.	YP_002956107.1	Human cosavirus A	
8.	YP_002268402.1	Seneca valley virus	Senecavirus
9.	NP_740434.1	Theilovirus	Cardiovirus
10.	YP_001950232.1	Human TMEV-like cardiovirus	
11.	YP_001816892.1	Saffold virus	
12.	NP_740359.1	Porcine teschovirus	Teschovirus
13.	ADN94255.1	Turkey hepatitis virus 2993D	
14.	ADI48258.1	Bat kobuvirus TM003k	
15.	YP_003853309.1	Turdivirus 2	
16.	YP_003853298.1	Turdivirus 1	
17.	AEA03667.1	Picornavirus chicken/CHK1/USA/2010	
18.	NP_740444.1	Aichi virus 1	Kobuvirus
19.	YP_002473944.1	Porcine kobuvirus	
20.	NP_859028.1	Aichivirus B	
21.	YP_004564619.1	Pigeon picornavirus B	
22.	YP_164831.1	Avian sapelovirus	
23.	NP_740525.1	Human rhinovirus B14	Enterovirus
24.	AAW83322.1	Human coxsackievirus B3	
25.	YP_001718564.1	Simian enterovirus 43	
26.	YP_004782555.1	Bat picornavirus 1	
27.	YP_005352655.1	Canine picornavirus	
28.	NP_937979.1	Simian sapelovirus 1	Sapelovirus
29.	NP_740489.1	Porcine sapelovirus 1	
30.	NP_740559.1	Hepatitis A virus	Hepatovirus
31.	NP_705605.1	Avian encephalomyelitis virus	
32.	AGH06056.1	Turkey avisivirus	
33.	YP_001497184.1	Seal picornavirus type 1	Aquamavirus
34.	AFH54140.1	Duck hepatitis A virus 1	Avihepatovirus
35.	ABO09966.1	Duck hepatitis virus 2 strain 90D	
36.	NP_705884.1	Ljungan virus	Parechovirus
37.	ABS82469.1	Human parechovirus 1	
38.	YP_006628187.1	Bluegill picornavirus	Piscevirus (proposed)

## Results

### Virus Isolation

**Figure 1:**
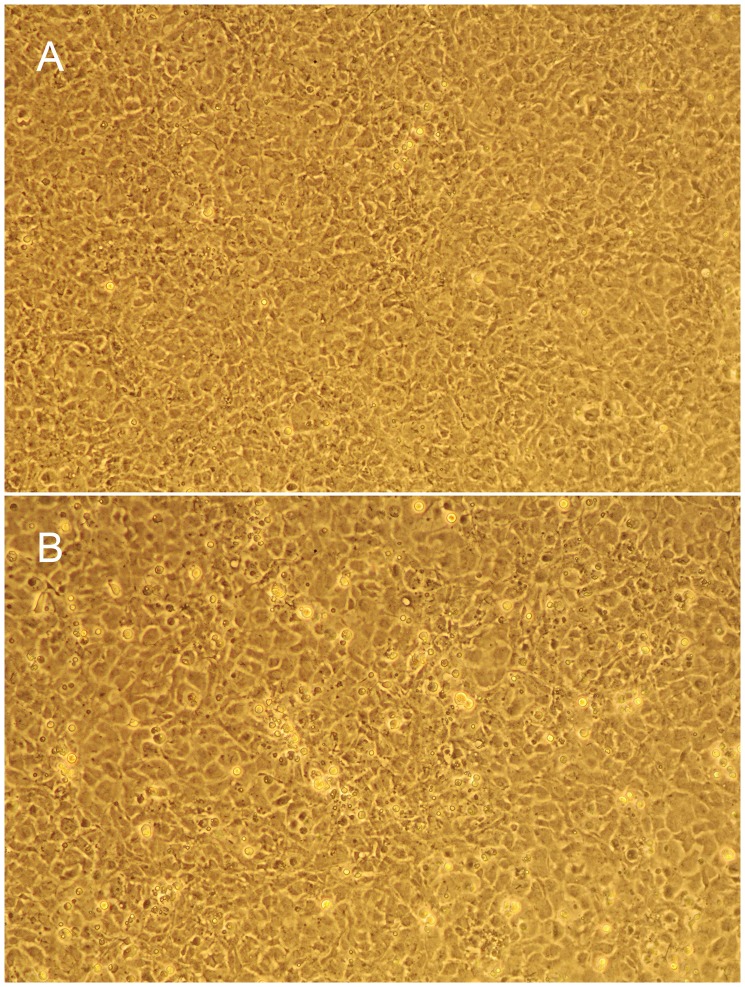
Fathead minnow picornavirus infected cell culture. A) Healthy epithelioma papulosum cyprini cells (EPC), B) EPC cells incubated at 22°C, 6 days post-inoculation with fathead minnow picornavirus (FHMPV-09).

Twenty samples from fathead minnows and brassy minnows exhibited CPE five to six days after inoculation on the EPC, FHM, CHSE-214, BF-2, or RTG-2 cell line at 15–22°C (Figure 1). The CPE was characterized by rounding and aggregation of cells, with eventual widespread epithelial cell sloughing. For FHMPV-01, a total of four passages were made and the CPE was consistent and present at each successive passage. No plaque assay was performed and the virus was not titrated. No other viral agents were isolated by cell culture. Six of the FHMPV isolates originated from fish with ocular and dermal hemorrhage and the remaining 14 from apparently healthy fish.

### Morphologic Characterization

**Figure 2:**
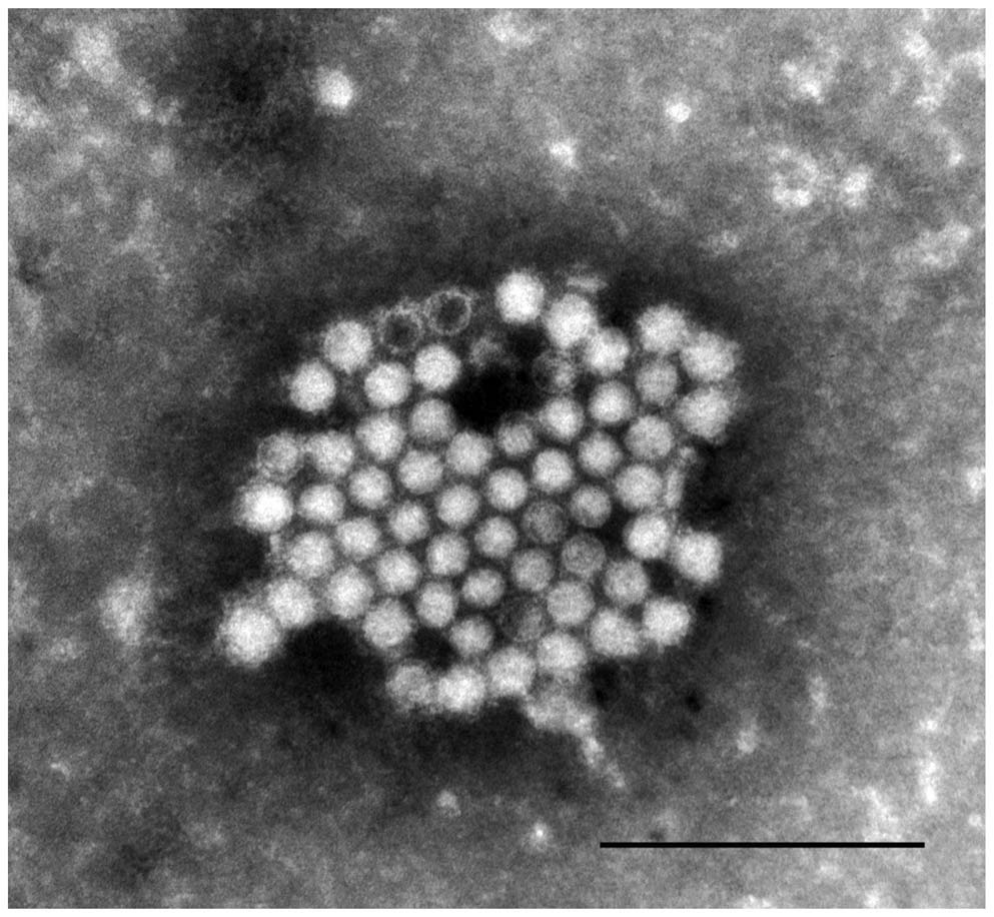
Electron micrograph of fathead minnow picornavirus. Negative contrast electron microphotograph of FHMPV-03 showing aggregation of non-enveloped spherical (∼ 30–32 nm) virions consistent with virus of the *Picornaviridae* family. Bar = 200 nm.

Negative contrast electron microscopy of four of the isolates revealed the presence of featureless, non-enveloped, spherical, ∼30–32 nm virus particles (Figure 2) similar to those described for members of the family *Picornaviridae*.

### Molecular Characterization

By amplification and sequence analysis, FHMPV was observed in all CPE positive cell cultures but not in mock-inoculated negative control cells. BLASTx analysis of sequences obtained from partial genome sequencing provided a match with members of the *Picornaviridae* family.

The complete genome of the FHMPV-09 isolate was 7834 nucleotides in length and contained a single ORF encoding a polyprotein of 2311 amino acids (Table 3). This positive sense RNA virus genome began with a 5′ NTR of 557 nt, followed by the 6933 nt ORF (558–7490), a 3′ NTR of 344 nt, and concluding with a long poly (A) tail. The complete genome of this isolate was submitted to GenBank as accession KC465953.

**Table 3:** Prediction of different polyproteins in FHMPV genome.

	Nucleotide sequence			Amino acid sequence			
Gene	Start	End	Size	Start	End	Size	Predictedcleavage site
5′ NTR	1	557	557				
VPO	558	1274	717	1	239	239	
VP3	1275	1970	696	240	471	232	Q/G
VP1	1971	2768	798	472	737	266	Q/G
P1	558	2768	2211	1	737	737	
2A1	2769	2867	99	738	770	33	Q/G
2A2	2868	3263	396	771	902	132	NPG/P
2A3	3264	3593	330	903	1012	110	NPG/P
2B	3594	3992	273	1013	1145	133	E/S
2C	3993	4991	1125	1146	1478	333	E/D
P2	2769	4991	2223	738	1478	741	
3A	4992	5255	264	1479	1566	88	Q/A
3B	5256	5345	90	1567	1596	30	E/A
3C	5346	5948	603	1597	1797	201	Q/G
3D	5949	7487	1539	1798	2310	513	Q/G
P3	4992	7487^a^	2496	1479	2310	832	
3′ NTR	7491	7834	344				

a 7488–7490: Stop codon (TGA).

### Polyprotein of FHMPV-09

**Figure 3:**
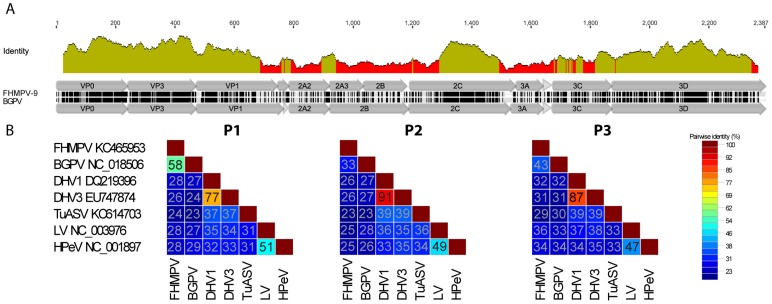
Fathead minnow picornavirus genome and diversity. Sequence divergence of FHMPV with other picornaviruses. A) Sequence identity plot comparing the complete polyprotein of FHMPV-09 and BGPV (YP_006628187.1), green represents amino acid identity between 30%–100% and red represents identity less than 30%. B) Pairwise comparisons of closely related picornaviruses in the P1, P2 and P3 regions. Each number represents the pairwise amino acid identities between the corresponding species.

The P1 segment was 737 aa long. It encodes for capsid proteins VP0, VP3, and VP1. VP0 was not cleaved into VP4 and VP2, similar to human parechovirus (HPeV), Ljungan virus (LV) and duck hepatitis virus (DHV). VP0 was predicted to be 239 amino acids (557–1273 nt position) in size and gave maximum identity (64%) with BGPV on BLASTx analysis followed by HPeV, LV and DHV. No consensus sequence GXXX(S/T) responsible for myristoylation was identified. In the VP3 protein of FHMPV-09, a stretch of 22 aa (GRFAVFVLNPLTYTPACPSAVR) at the 426 to 447 amino acid position was identical with that found in BGPV. In addition, the conserved amino acid sequence (VLNPLTYT) was found in VP3, which is very similar to V(L/V)NRT(Y/V/F)N sequence found in HPeV, LV and DHV. Among all proteins, VP3 of FHMPV shared the highest identity with BGPV (Figure 3). The cleavage site Q_471_/G_472_ is predicted to be the start point of VP1 protein and is 266 aa in length. The integrin binding arginine-glycine-aspartic acid (RGD) motif was absent in VP1 protein of FHMPV-09.

The cleavage site Q/G at position 737/738 is predicted to be the start of the P2 segment, which was 741 aa in length and encoded for non-structural proteins 2A, 2B and 2C. The 2A protein is further divided into 3 proteins (2A1, 2A2 and 2A3) of which 2A1 is 33 aa long and has the conserved sequence CGDVESNPGPD with G|P, which is considered the junction of 2A1 and 2A2. 2A2 was 132 aa in size and had conserved motif SGDVEQNPGPV. 2A3 begins at the second NPG|PV of the previous protein and extends 110 aa in length. In the 2A protein of FHMPV, the H-box and NC motif were not present. The 2B protein is predicted to be 133 aa in length. Comparison with BGPV, many insertion and deletions in the 2A and 2B region of FHMPV-09 were found (Figure 3). The 2C protein begins at position 1145 (cleavage site E/D) and is 333 aa in length. The 2C sequence contained the conserved NTPase motif (G/S)XXGXGK(S/T) [39].

Segment P3 contains non-structural proteins 3A, 3B, 3C and 3D. The 3A protein is 88 aa in length and did not match with picornavirus sequences on BLASTx analysis. The 3B protein is the smallest (30 aa) and has a tyrosine (Y) residue at the 3^rd^ position. In the 3C protein, the conserved sequence GDCGS was present, similar to GXCG(G/S) in most picornaviruses. Based on amino acid alignment with DHV, LV, and HPeV, a catalytic triad was predicted: histidine (H), aspartic acid (D), and cysteine (C) at positions 42, 78, and 154 of the 3C protein, respectively. The protein 3D (513 aa) coding for RdRp contained KDELR, DYS, PSG, YGDD and FLKR motifs, in which DYS, PSG, and YGDD are the part of polymerase site of the 3D protein.

### Sequence Comparisons and Phylogenetic Analysis

**Figure 4:**
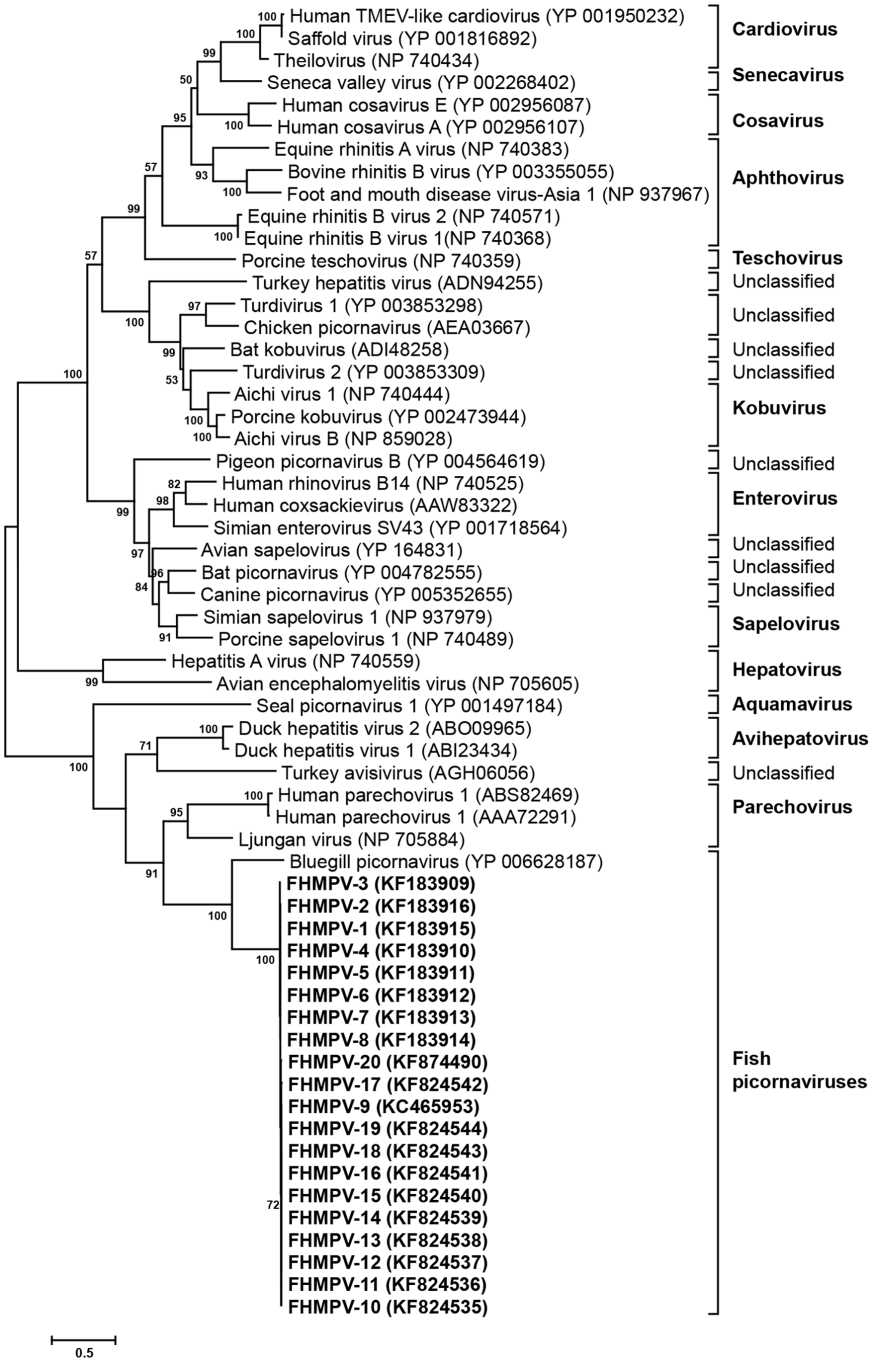
Phylogenetic analysis of picornavirus 3D gene sequences. Phylogenetic analysis on the basis of amino acid sequence of complete picornavirus 3D gene. The evolutionary history was inferred using the Maximum likelihood method in MEGA5. The percentage of replicate trees in which the associated taxa clustered together in the bootstrap test (1,000 replicates) are shown next to the branches. The evolutionary distances were computed using the JTT+G+I method.

Sequence comparisons based on the 491 amino acid sequence of the 3D gene showed that all isolates of this study had 98.6% to 100% identity among themselves but only 49.5% identity to the 3D gene of BGPV. Comparison with picornaviruses from other species indicated identity of 43.6% with LV and DHV-1, while only 36.0% with human parechovirus (Figure 4). The amino acid sequence analysis predicted the presence of highly conserved 3D sequences KDELR, DYS, PSG, YGDD and FLKR in all isolates. This novel picornavirus has been tentatively named the fathead minnow picornavirus (FHMPV). Although all isolates analyzed in this study were closely related, when analyzing single nucleotide polymorphisms among the isolates, 95 synonymous and 2 non-synonymous nucleotide changes were found. These changes differentiate isolates FHMPV-01 thru -08 (from Minnesota and Wisconsin) from FHMPV-09 thru -19 (from Montana) and FHMPV-20 (from Illinois). In FHMPV isolates from Minnesota and Wisconsin, the amino acids threonine and glutamic acid were predicted at positions 95 and 394 of the 3D gene, compared to serine and aspartic acid in the Montana and Illinois isolates. In addition, there was an alanine residue in FHMPV-01 thru -19 at position 149, while a valine residue was predicted at this site in FHMPV-20. At position 476, a serine was predicted in FHMPV-01 thru -08 and FHMPV-20 while asparagine was predicted in FHMPV-09 thru -19.

**Figure 5:**
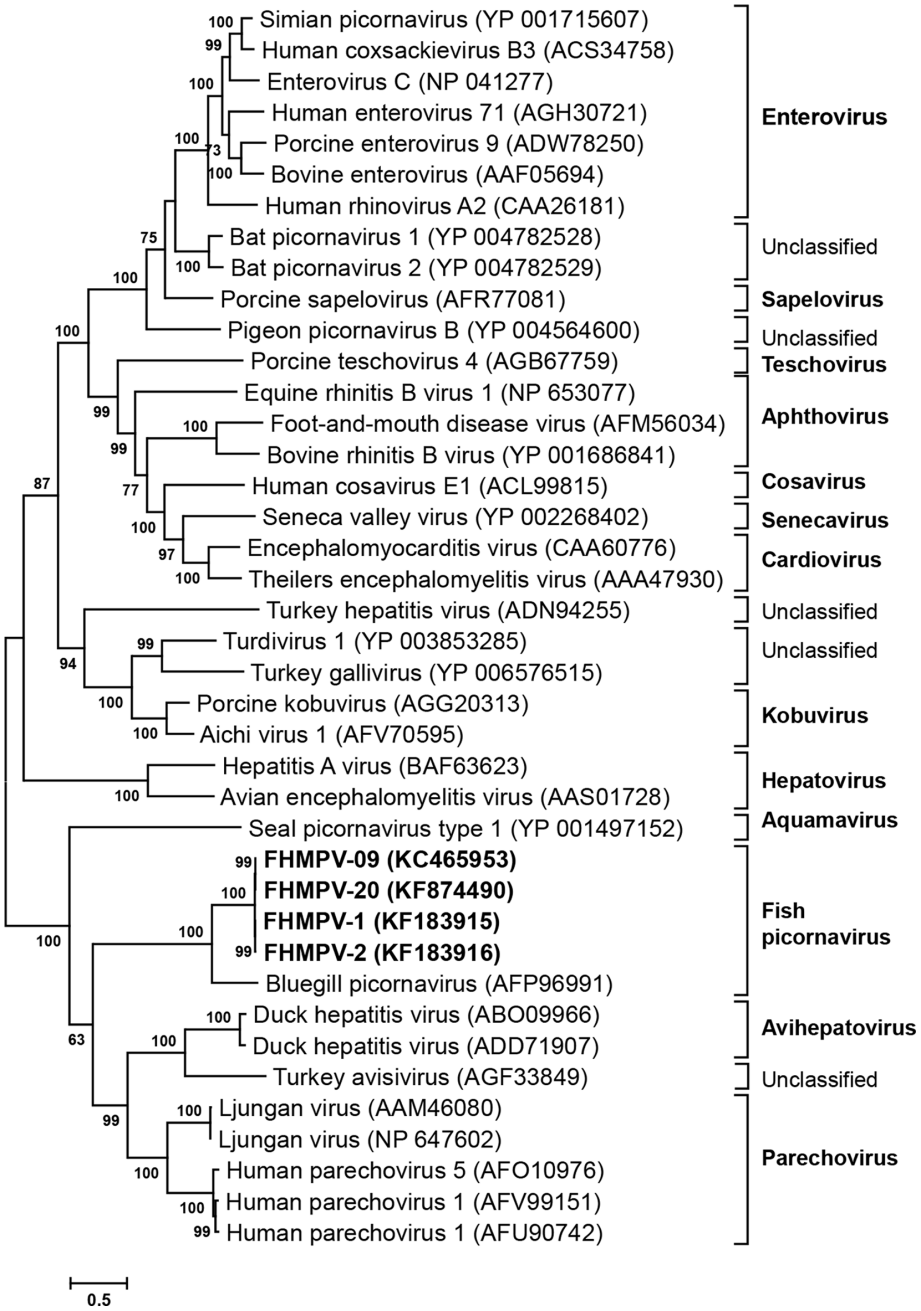
Phylogenetic analysis of picornavirus complete genome sequences. Phylogenetic analysis on the basis of complete genome (open reading frame) amino acid sequence of picornavirus. The evolutionary history was inferred using the Maximum likelihood method in MEGA5. The percentage of replicate trees in which the associated taxa clustered together in the bootstrap test (1,000 replicates) are shown next to the branches. The evolutionary distances were computed using the JTT+G+I and are in the units of the number of amino acid substitutions per site.

Pairwise comparison of the entire polyprotein of FHMPV-9 and the BGPV (Genbank NC018506) resulted in 48.1% nucleotide and 42.0% amino acid identity (Figure 5). FHMPV shared 58% (P1), 33% (P2) and 43% (P3) amino acid identities with BGPV (Figure 3). Both BGPV and FHMPV shared less than 40% identities in all P1, P2, P3 regions with other picornavirus genomes (Figure 3).

## Discussion

In this study, we report the isolation, morphology, and molecular characterization of FHMPV, a novel picornavirus of baitfish. Highly conserved 3D sequences were found within the FHMPV isolates from this study, which is a characteristic of the *Picornaviridae* family [23], [27]. FHMPV shared only a 49.5% amino acid identity with BGPV, the nearest neighbor.

According to criteria proposed by the International Committee on Taxonomy of Viruses (ICTV), members of the same picornavirus genus should share greater than 40%, 40% and 50% amino acid identities for P1, P2, and P3 regions, respectively. The sequence identities between FHMPV and BGPV of 58% (P1), 33% (P2) and 43% (P3) and the fact that both viruses share less than 40% sequencing identify with members of other genera indicate that FHMPV and BGPV could be considered prototypes of two new genera of *Picornaviridae*. We propose the novel genus name *Piscevirus* to reflect the host (Latin translation of fish is Pisces) of FHMPV, a second picornavirus genus of fish.

The family *Picornaviridae* is very diverse having numerous genera and serotypes. Hence, it is not surprising to detect more than a single picornavirus among fish species. However, very little is known about picornaviruses in fish. Together with the recent discoveries of other fish picornaviruses [20], [23], the picornavirus host range is further expanded among ray-finned fishes (class *Actinopterygii*). Although in the same class *Actinopterygii*, fathead minnow, bluegill, and European eel belong to three different orders, *Cypriniformes*, *Perciformes*, and *Anguilliformes*, respectively. The broad characterization of picornaviruses in fish provides evidence that picornavirus evolution may antedate the radiation of vertebrate species including fish, reptile, amphibian and mammal, perhaps in a “big bang” fashion similar to that of the *Picornavirales* Superfamily [40]. Given that the known fish picornaviruses are highly divergent, it suggests that a high diversity of picornavirus is yet to be characterized in fish species.

The complete polyprotein of FHMPV divided into three segments P1, P2 and P3 out of which P1 contains structural proteins while P2 and P3 contains non-structural proteins. In segment P1, the first protein, VP0, does not have the conserved sequence GXXX(S/T) for myristoylation, which has been observed mainly in avian encephalomyelitis virus, hepatitis A virus, HPeVs and LV. Myristoylation involves the covalent linkage of myristic acid to an N-terminal glycine residue in consensus GXXX(S/T) motif, which plays an important role in virion morphogenesis. However, the finding is in correlation with the DHVs and BGPV, which also do not have this motif [23], [41], [42]. It has been suggested that, for hepatoviruses, the blocked N-terminus of VP0 possibly indicates an alternative modification that substitutes for the myristate motif [43]. Thus, a similar hypothesis could be proposed for the FHMPV. The VP3 protein was found to be the most conserved among all proteins to BGPV, containing a long stretch of 22 identical amino acids. The significance of this is not known at this time. The absence of an integrin binding RGD motif in the VP1 is similar to the absence of this receptor in DHV-1, HPeV3 and LV. This motif is known to mediate many cell to cell and microbe-host interaction, hence the absence of this motif implies the existence of an alternative cell receptor in FHMPV, which needs to be studied in the future. It is intriguing that between FHMPV and BGPV, the P1 region that encodes for structural proteins is more conserved than the P2 and P3 regions that encode for non-structural proteins such as helicase and RdRp. One possible explanation might be a greater degree of conservation among viral capsid sequences needed for the infection of fish.

Like DHVs, the 2A protein is likely divided into 3 proteins (2A1, 2A2, 2A3), with the presence of two conserved sequences CGDVESNPG|PD and SGDVEQNPG|PV. The NPG|P seems to be the junction of 2A1|2A2 and 2A2|2A3. The conserved motif in 2A2 matched with human cosavirus and BGPV, with a conserved motif RLXLLLSGDXEXNPGP and SGDVEQNPGPX respectively. The proposed presence of 2A3 in FHMPV-09 genome was determined based on the presence of amino acid sequence between second NPG|P and 2B. A similar prediction was made by Barbknecht [23] for the BGPV genome. The conserved H-box and NC motifs that are considered to control cell proliferation [44] were absent in FHMPV. These motifs are mainly present in DHVs and HPeVs, but also absent in newly reported swine pasivirus 1 [42]. The 2A protein was found to be divergent, containing insertion and deletions compared to BGPV, and should be the focus of future study to better understand the function of this protein. The 2C protein displayed the NTPase motif (G/S)XXGXGK(S/T) that is present in all picornaviruses. In the P3 segment, 3A and 3B proteins have less identity with other picornaviruses. 3B have tyrosine residue which is required for the attachment of VPg protein to the 5′ NTR uracil of RNA genome which act as an RNA replication primer as suggested by Ambros and Baltimore [45]. In 3C, a conserved motif GDCGS is present that correspond to GXCG(G/S) motif present in all picornaviruses. The catalytic triad H42-D78-C154 is very close to H41-D79-C154 catalytic triad of LV. In the 3D protein, like all members of *Picornaviridae* family that have KDE(I/L)R, DYS, (P/C)SG, YGDD and FLKR conserved motif at the carboxyl end of the polyprotein, FHMPV also has the five conserved motifs (KDELR, DYS, PSG, YGDD and FLKR).

The 18 isolates from fathead minnows and two from brassy minnows were highly similar (98.6–100%), suggesting a rapid and widespread dissemination. Many of these isolates were collected from baitfish dealers, who were collecting fish from a variety of sources and distributing them across a wide geographic range. For example, it is possible for farm raised baitfish from South Dakota to be mixed with wild harvested baitfish from Minnesota and farm raised baitfish from Arkansas at a Wisconsin baitfish dealer, and distributed to any other number of states. As the name suggests, baitfish are often used for angling and have the potential to be directly introduced into susceptible populations. While hemorrhagic lesions were observed in some fish from which the virus was isolated, the two cannot be conclusively linked without a more thorough study of viral pathogenesis.

While FHMPV may not induce mortality in fathead minnows, susceptibility of other farmed and wild predatory species is unknown. In the report on BGPV, there appears to be association with hemorrhage on the skin, erythema, pale and swollen internal organs, and mortality [23]. Diagnostic investigation, albeit limited, of farmed and wild fish populations has not linked clinical lesions in predatory species with FHMPV. Consequently, the long-term impact to the health of fish populations is currently unknown. However, the high level of FHMPV prevalence in the stocks we tested demonstrate that the unregulated movement of ornamental and baitfish species could serve as important pathways for introduction of exotic pathogens, some of which may present significant levels of risk to native aquatic species. Given the importance of these host species to the economy and ecology of the region, continued research is necessary.
